# Integrative Genomic Mapping and Visualization From Curated Public Datasets Reveals Germline *RB1* Variant Diversity in Retinoblastoma

**DOI:** 10.1167/iovs.67.8.40

**Published:** 2026-07-16

**Authors:** Elizabeth Rooks, Matthew Lee, Oieswarya Bhowmik, Viridiana Hernandez-Lopez, Andrew W. Stacey, Debarshi Mustafi

**Affiliations:** 1 Department of Ophthalmology, University of Washington, Seattle, Washington, United States; 2 Roger and Angie Karalis Johnson Retina Center, Seattle, Washington, United States; 3 Kaiser Permanente Bernard J. Tyson School of Medicine, Pasadena, California, United States; 4 Fred Hutchinson Cancer Consortium, Seattle, Washington, United States; 5 Division of Ophthalmology, Seattle Children's Hospital, Seattle, Washington, United States; 6 Brotman Baty Institute for Precision Medicine, Seattle, Washington, United States

**Keywords:** PANORAMA, retinoblastoma, RB1, variant visualization

## Abstract

**Purpose:**

To consolidate and refine *RB1* disease-causing variants in retinoblastoma (RB) and to develop a visual, integrative tool for variant exploration at the gene and protein level.

**Methods:**

*RB1* variants associated with RB were compiled from the Leiden Open Variation Database, 13 published cohort studies between 2014 and 2025, and the Catalogue of Somatic Mutations in Cancer. Case information was recovered where available, and variants were reannotated using Human Genome Variation Society (HGVS) nomenclature on transcript NM_000321.3 and mapped to Genome Reference Consortium Human Build 38 (GRCh38). PANORAMA was built in Python using Dash and Plotly.

**Results:**

After filtering for variants with unique identifiers and interpretable descriptions, 3004 variants (1943 germline, 797 somatic, and 264 variants of unknown origin) underwent HGVS standardization, GRCh38 mapping, and statistical analysis. Truncating germline variants accounted for more than 80% of variants, whereas missense variants comprised 9.2%. Germline variant density was highest in exons 4 and 15 and lowest in exons 25 to 27. Nonsense variants predominated in the N-terminal, Pocket A, and C-terminal regions, whereas missense variants were concentrated in Pocket B. Among 1539 germline variants with known tumor laterality, 81.2% were bilateral. Only 39.4% of germline variants were reported in ClinVar. PANORAMA allowed interactive visualization and filtering of aggregated and user-uploaded *RB1* variants.

**Conclusions:**

This work consolidates dispersed *RB1* variant data into an unified resource and reveals domain-specific patterns of genetic disruption in RB. Integration with PANORAMA supports exploratory analysis, variant interpretation, and comparison of external cohorts to the global *RB1* landscape.

Retinoblastoma (RB) is the most common pediatric intraocular malignancy and has served as a foundational model in cancer biology. Knudson's epidemiological study of RB patients established the “two-hit” hypothesis, introducing the concept that loss of a tumor suppressor gene could drive oncogenesis.^1^ Fifteen years later, cloning of the causative RB gene, *RB1*, on chromosome 13 provided molecular validation of this model.^2^^,^^3^ The *RB1* gene product, retinoblastoma protein (pRB), has since been biochemically characterized as a 928-amino-acid nuclear phosphoprotein that regulates cell-cycle progression.^3^^–^^5^ The loss of this key regulatory protein results in unchecked proliferation of cells and tumor initiation. Importantly, those who harbor a germline *RB1* variant have a lifelong risk of secondary malignancies with significant morbidity and mortality.^6^^–^^8^

Advances in sequencing technologies have accelerated our understanding of the variants that drive *RB1*-associated oncogenesis. Deposition of data in gene variant databases and those reported in individual publications have uncovered thousands of disease-causing variants in *RB1*. These data have demonstrated the distribution of variants throughout the 27 exons encoded by the 180-kilobase (kb) locus and have indicated the potential presence of regional hotspots in *RB1*.^9^^–^^12^ Whereas certain variant classes, such as nonsense or frameshift mutations, can be predicted to be pathogenic as they disrupt protein function, the effects of hypomorphic variants, deep intronic variants, modifier genes, and imprinting on protein expression reflect the complexity of the *RB1* variant landscape that underlies this cancer syndrome.^13^^–^^15^

Exploring this variant diversity is a challenging task because *RB1* variants are dispersed across sources and often lack variant level details for consolidation. The Leiden Open Variation Database (LOVD2), together with its recently converted genome-centric version (LOVD3),^16^^,^^17^ represent the most comprehensive catalog of *RB1*-causative variants in both germline and somatic cases. The Catalog of Somatic Mutations in Cancer (COSMIC) provides further insight into somatic *RB1* variation resulting from RB tumor sequencing. Cohort studies of RB aiming to genetically and clinically characterize populations also report both germline and somatic *RB1* variants. The fragmentation of variant data across sources has hindered efforts to systematically assess genotype–phenotype correlations or identify regional patterns driving *RB1*-associated tumorigenesis.

To construct a repository of *RB1* genetic variants in patients with RB, we undertook an initiative to integrate variant datasets reported across LOVD, COSMIC, and peer-reviewed publications; standardize variants to the Human Genome Variation Society (HGVS) nomenclature; and map variants to the Genome Reference Consortium Human Build 38 (GRCh38), its corresponding Ensemble RNA transcript, and the UniProt protein reference. As the catalog of reported variants expands, dimensional visualization will be essential to detect patterns that remain obscured in tabular representations that predominate variant-level data. Unfortunately, existing tools to visualize variant data are limited, as many, such as Mutplot,^18^ MutationMapper,^19^ ProteinPaint,^20^ and g3viz,^21^ display variants in a proteomic context rather than genomic context, thereby limiting non-coding variant mapping. Others, such as GenVisR,^22^ Variant Visualization (VIVA),^23^ and vcfR,^24^ display variants in a genomic context but lack protein context and have limited dynamic exploration of variants. Moreover, no current tool provides an accessible, interactive interface for variant cohort data exploration in both genomic and proteomic contexts as well as haplotype data for parent-of-origin exploration.

In this work, we curated *RB1* variant data from large variant repositories and published data, and we standardized their annotation to explore their genomic and proteomic contexts. To overcome the limitation of visualization tools to model and explore genomic and proteomic contexts, we developed Phased Allelic Navigation and Omic-Resolved Annotation, Mapping, and Analysis (PANORAMA). This tool enables variant exploration at the gene and protein sequence levels within the curated dataset while also allowing users to upload and interrogate their own datasets. By enabling filterable categories such as variant consequence, tumor laterality, and parent of origin, the tool provides insight into features that have limited our understanding of the genotype-to-phenotype relationship in RB. By unifying dispersed variant data into an accessible and dynamic interface, this work provides a consolidated view of *RB1* variation and supports deeper investigation into the variant architecture that drives RB.

## Methods

### Data Sources

Data were integrated from four sources: the legacy *RB1* locus-specific database (LOVD2), the genome-centric *RB1* database (LOVD3), 13 studies ranging from 2014 to 2025, and the COSMIC database (v102).

#### LOVD Databases

The *RB1* dataset within the LOVD3 database (v3.0, Build 30b) represents data transitioned from the earlier LOVD 2 database (*rb1-lsdb*, LOVD v2.0, Build 34). This migration was performed using a conversion script that employs VariantValidator and Mutalyzer to standardize and map variants to the GRCh38 human reference genome.^25^^,^^26^ We reconciled the LOVD2 and LOVD3 datasets to obtain case-level detail and capture multiple submitted instances of the same variant. The *RB1* LOVD2 website (http://rb1-lovd.d-lohmann.de) was accessed on September 2, 2025, and variants were manually retrieved. The *RB1* LOVD 3 website (https://databases.lovd.nl/shared/view/RB1) was accessed on October 8, 2025, and variants were downloaded as a tab-separated values (.tsv) file. Within the file, there were 12 separate dataframes because the database stores genomic, clinical, and variant information in separate linked tables. We re-associated the “genome,” “transcript,” and “individual” dataframes using the variant_ID and the individual_ID columns. Individual IDs were only assigned to 70 of the variants, as these were new entries since the creation of LOVD3.

LOVD2 and LOVD3 records were reconciled using the shared database IDs (DBIDs) and patient IDs. To note, the LOVD2 DBID format contained four numerical characters (RB1_####), and LOVD3 contains five (RB1_#####). These represent the same variants; therefore, LOVD2 DBIDs were adapted to match the LOVD3 format by adding a leading zero (RB1_0####). LOVD3 and LOVD2 entries were paired based on the DBID, and the cDNA change was cross-referenced to verify that the entries represented the variant. This resulted in a reconciled set of LOVD variants with case identifiers and phenotypic information.

#### Published Cohort Studies

A PubMed search was conducted using the terms “retinoblastoma,” “germline or hereditary,” “*RB1*,” and “variant or mutation,” limited to studies published in the last 20 years. We also reviewed the reference lists of retrieved articles to identify additional relevant studies. Studies were included if they reported all individual *RB1* variants found in a cohort of patients with clinically confirmed RB. Studies that deposited their data in LOVD, as determined from reviewing study methods and references listed within LOVD, were not included. Variant data were extracted from supplementary materials or were manually obtained from published tables. Variants that did not specify the exact coordinates of a large deletion were excluded, as they could not be mapped within the viewer. When reported, accompanying case data such as phenotype (unilateral versus bilateral disease) and variant origin (germline versus somatic) were included.

#### COSMIC

COSMIC (v102)^27^ was queried to identify RB tumor samples with *RB1* variants. The search was restricted to entries annotated under the primary site “eye” and histology “retinoblastoma” for variant inclusion.

### Data Curation

**Figure 1.**
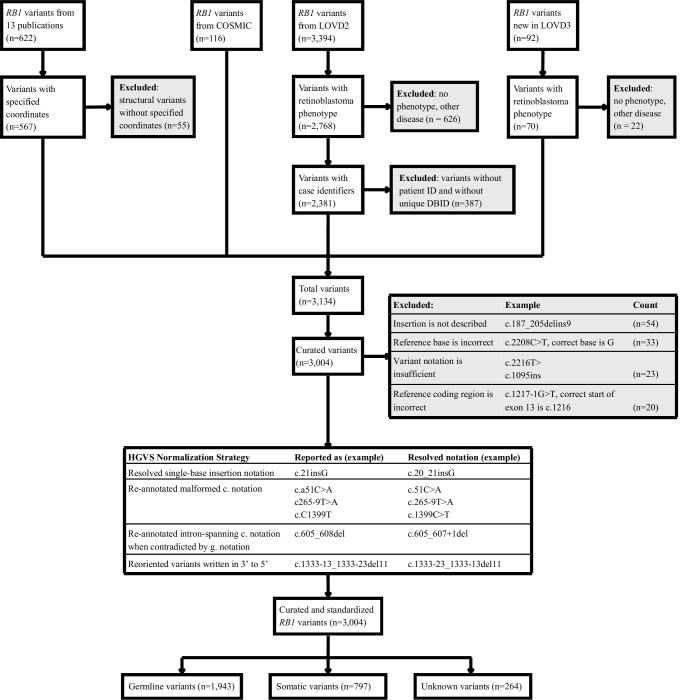
Workflow used to assemble and standardize the *RB1* variant dataset. Variants from COSMIC, published cohorts, and LOVD were filtered for RB phenotype, case identifiers, and notation that could be interpreted and converted to HGVS, yielding 3004 entries. HGVS normalization strategies are described with examples provided.

Data were reviewed and refined according to the workflow in Figure 1 to generate a curated and standardized dataset of *RB1* variants for downstream analysis. Within the LOVD2 and LOVD3 datasets, entries that did not specify the patient's phenotype as RB were removed. Patient IDs and DBIDs were standardized for analysis, and entries were assigned unique IDs in the format DBID_PatientID. Variants that lacked both a patient identifier and a unique DBID could not be confidently linked to an individual case and were excluded from analysis. Variants sourced from publications received a unique ID in the format PatientID_AuthorLastname. Variants sourced from COSMIC retained the original ID. The source publication PubMed reference numbers (PMIDs) associated with each COSMIC record were retrieved and cross-referenced against the PMIDs of the 13 cohort studies included in our analysis to eliminate any potential duplicate entries. Variants that could not be unambiguously interpreted from their coding nomenclature were excluded from analysis. All filtered variants were then manually reviewed to ensure that they were formatted according to the HGVS nomenclature standard.^28^ Variants were mapped onto the Matched Annotation from NCBI and EMBL-EBI (MANE) select transcript (NM_000321.3) on GRCh38 using liftOver (Broad Institute, Cambridge, MA, USA; March 3, 2024). This tool employs the UCSC Genome Browser liftOver command-line tool for intervals and positions and a bcftools plugin for variants to generate normalized genomic coordinates that are left aligned and parsimonious.^29^ The liftOver results for 336 required manual review and were checked against the transcript in the UCSC Genome Browser for notational accuracy. Entries that had a variant origin of “unknown” were reclassified as germline if there was a family history of RB, if the variant was identified in peripheral blood, or if the variant was described as mosaic. Annotations within the remarks or the originated_in columns were used to further variants of unknown origin as germline or somatic. Paternal or maternal inheritance was obtained from these columns as well or was identified in published cohorts.

Computational impact scores, populational allele frequencies, and ClinVar pathogenicity assertions were acquired to support variant interpretation. Combined Annotation Dependent Depletion (CADD) scores were generated by submitting variants as a variant call format (VCF) file to the CADD web server (v1.7) and exporting the resulting .tsv file.^30^ Missense variant pathogenicity scores were obtained from the precomputed AlphaMissense dataset.^31^ The synVep predictions for synonymous variants were obtained by querying each variant and downloading the corresponding .csv file.^32^ Allele frequency data were obtained from gnomAD (v4.1.0) by accessing the *RB1* gene page and downloading the .csv file. ClinVar classifications for *RB1* were retrieved by querying the gene and exporting the associated .tsv file. Annotated molecular consequence (e.g., frameshift, nonsense, splice) was compared to the classification of the variant in ClinVar when available, and differences were resolved following American College of Medical Genetics and Genomics (ACMG) rules from the 2015 guideline and by mapping on the transcript within the UCSC genome browser.^33^ Thirty-one variants that were unlikely to be pathogenic due to higher than an expected minor allele frequency of 3.7 × 10^−6^ (based on penetrance, disease prevalence, and maximum allelic contribution) were flagged in red so they could be visually distinguished in PANORAMA.

### PANORAMA Variant Viewer Coding

PANORAMA was built in Python 3.9 using the packages Dash and Plotly for the web interface and visualization and pandas for data handling. Curated *RB1* genomic variants mapped to GRCh38 were imported and aggregated at the level of unique genomic coordinates and constitute the default dataset within PANORAMA. The tool also allows users to upload their own variants for local visualization within the tool. Accepted file types include .csv, .tsv, and .xlsx, with data formatted as four columns (chromosome, position, reference, and alt) or one column with hyphen-delimited or colon-delimited coordinates. The main panel is a scalable lollipop plot of variants across the *RB1* locus, with the number of reported variants on the *y*-axis. Variants are colored by data source, and hovering over a point reveals the variant description, the number of reports, and the source. The second aligned panel is a gene map of *RB1* with hover text to identify the exon, intron, and protein domain. Exon and intron regions were assigned using coding sequence intervals retrieved from the Ensembl REST application programming interface (API) using transcript version ENST00000267163. Protein domains of pRB were assigned using protein positions obtained from the UniProt API using ID P06400, and were mapped to genomic coordinates. The web interface provides filters for data source (COSMIC, LOVD, publications); molecular consequences (3′UTR, 5′UTR), including promoter variants, frameshift, inframe indel, inframe insertion, missense, synonymous, splice, splice acceptor, splice donor, start lost, nonsense, intron, large deletion; genetic origin (germline, somatic, unknown); laterality (bilateral, unilateral, unknown); and ClinVar pathogenicity (pathogenic, pathogenic/likely pathogenic, likely pathogenic, likely benign, benign/likely benign, benign, conflicting classifications of pathogenicity, uncertain significance). Dash callbacks are used to dynamically re-render the plots in response to filter changes or file uploads and to generate a downloadable comma-separated value (CSV) file corresponding to the filtered dataset. Additionally, when a user-uploaded file contains parent-of-origin information in the form of familial data or haplotype-specific methylation calls, PANORAMA shows an additional lollipop plot with a maternal and a paternal allele track. Variants in red represent methylated, or maternal, alleles, and variants in blue represent unmethylated, or paternal, alleles at the corresponding *RB1* genomic positions, providing a visual summary of allele-specific methylation across the genome obtained from long-read sequencing studies.^34^ Those with familial data but lacking methylation data are shown in gray. The viewer can be accessed at https://www.mustafilab.org/panorama.

The Mustafi Lab website will host PANORAMA. Curated *RB1* variant data will be reviewed and updated approximately every 6 months, and the most recent date of data revision will be posted on the landing page of PANORAMA. User-uploaded variant files are used only for the active visualization session and are not retained server-side. Access to the source code for PANORAMA can be obtained with a Data Usage Agreement.

### Statistical Analysis

Analyses were performed with the statistical programming language R 4.5.1 (R Foundation for Statistical Computing, Vienna, Austria). Descriptive statistics were used to summarize variant origin, type, molecular consequence, and clinical associations. Categorical variables were compared using Fisher's exact or χ^2^ tests. Two-by-two contingency tables were generated for individual variants, and Fisher's exact tests were performed to evaluate for a difference in laterality. As this analysis involved testing of multiple hypotheses across all variants, *P* values were adjusted using the Bonferroni correction method. A significance threshold of adjusted *P* < 0.05 was applied. A CADD score of 20 was used as the threshold for high predicted pathogenicity based on model calibration benchmarks.^30^^,^^35^

## Results

Disease-causing *RB1* variants were extracted from LOVD, COSMIC, and 13 published RB cohorts^10^^,^^36^^–^^45^ (Table 1). Of the 3486 *RB1* variants in LOVD, after excluding variants lacking phenotypic data, those associated with non-RB malignancies, and those missing unique patient identifier information, 2451 variants were included for further analysis. These were combined with 116 variants from COSMIC and 567 variants from published cohorts, resulting in a total of 3134 variants. After excluding 130 variants that lacked sufficient genomic detail for mapping, 3004 variants were standardized using HGVS nomenclature. Of these, 1943 (64.7%) were germline, 797 (26.5%) were of somatic origin, and 264 (8.8%) were of unknown origin (Fig. 1; Table 2).

**Table 1.** Variants Identified in Publications Included in Data Cohort

Study	Country	Patients, *n*	Variants, *n*
Price et al.^10^	United Kingdom	111	111
Lan et al.^39^	China	75	75
Tomar et al.^43^	Singapore	51	62
Lee et al.^40^	Korea	60	60
Akdeniz Odemis et al.^36^	Turkey	57	57
Xie et al.^44^	China	56	54
Chai et al.^37^	China	54	54
Linh et al.^57^	Vietnam	36	44
Devarajan et al.^38^	India	28	30
Mendes et al.^41^	Brazil	20	23
Rojanaporn et al.^42^	Thailand	18	18
Stacey et al.^34^	United States	16	16
Zheng et al.^45^	China	14	18
Total	—	596	622

**Table 2.** Characteristics of Germline and Somatic Variants

Category	Germline, *n* (%)	Germline Mosaic, *n* (%)	Somatic, *n* (%)	Unknown, *n* (%)	Total, *n* (%)
Sources					
LOVD	1609 (83.5)	16 (100.0)	609 (76.4)	98 (37.1)	2332 (77.6)
Publications	318 (16.5)	0	74 (9.3)	166 (62.9)	558 (18.6)
COSMIC	0	0	114 (14.3)	0	114 (3.8)
Laterality					
Bilateral	1244 (64.6)	6 (37.5)	70 (8.8)	58 (22.0)	1378 (45.9)
Unilateral	279 (14.5)	10 (62.5)	587 (73.7)	85 (32.2)	961 (32.0)
Unknown	404 (21.0)	0	140 (17.6)	121 (45.8)	665 (22.1)
Family history					
Yes	419 (21.7)	0	6 (0.8)	0	425 (14.1)
No	1071 (55.6)	15 (93.8)	522 (65.5)	47 (17.8)	1655 (55.1)
Unknown	437 (22.7)	1 (6.2)	269 (33.8)	217 (82.2)	924 (30.8)
Molecular consequence					
Nonsense	642 (33.3)	3 (18.8)	414 (51.9)	99 (37.5)	1158 (38.5)
Frameshift	571 (29.6)	5 (31.2)	205 (25.7)	82 (31.1)	863 (28.7)
Splice site	335 (17.4)	7 (43.8)	106 (13.3)	33 (12.5)	481 (16.0)
Missense	207 (10.7)	1 (6.2)	37 (4.6)	30 (11.4)	275 (9.2)
Intronic	81 (4.2)	0	12 (1.5)	11 (4.2)	104 (3.5)
Inframe	34 (1.8)	0	11 (1.4)	1 (0.4)	46 (1.5)
Synonymous	24 (1.2)	0	9 (1.1)	4 (1.5)	37 (1.2)
5′UTR	25 (1.3)	0	1 (0.1)	3 (1.1)	29 (1.0)
Large deletion	4 (0.2)	0	2 (0.3)	0	6 (0.2)
Start lost	3 (0.2)	0	0	1 (0.4)	4 (0.1)
3′UTR	1 (0.1)	0	0	0	1 (0.0)
Total	1927 (64.1)	16 (0.5)	797 (26.5)	264 (8.8)	3004

**Figure 2.**
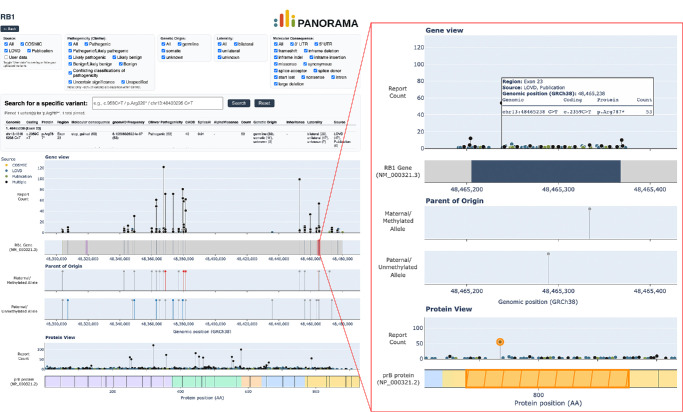
The PANORAMA interface displays the distribution of *RB1* variants across genomic and protein transcripts (*left panel*). The RB1 gene bar displays the exonic (*dark blue*) and intronic (*gray*) structure of RB1 with annotated features including the promoter region and the differentially methylated region (*purple*). The pRB protein bar displays the functional regions of the protein in different colors. Filters for variant source, pathogenicity, genetic origin, laterality, and molecular consequence are provided. Searching for a variant provides additional details and pins it within the genomic view (*white box*) and highlights it in the protein view (*orange*) as shown in the *right panel*. Also shown on the *right* is how the genomic and protein tracks can be interactively rescaled to examine variants at higher resolution. Hovering over a datapoint within the gene and protein views displays the exon or intron, functional region, genomic position, coding and protein-level annotations, and the count of variants at that position.

**Figure 3.**
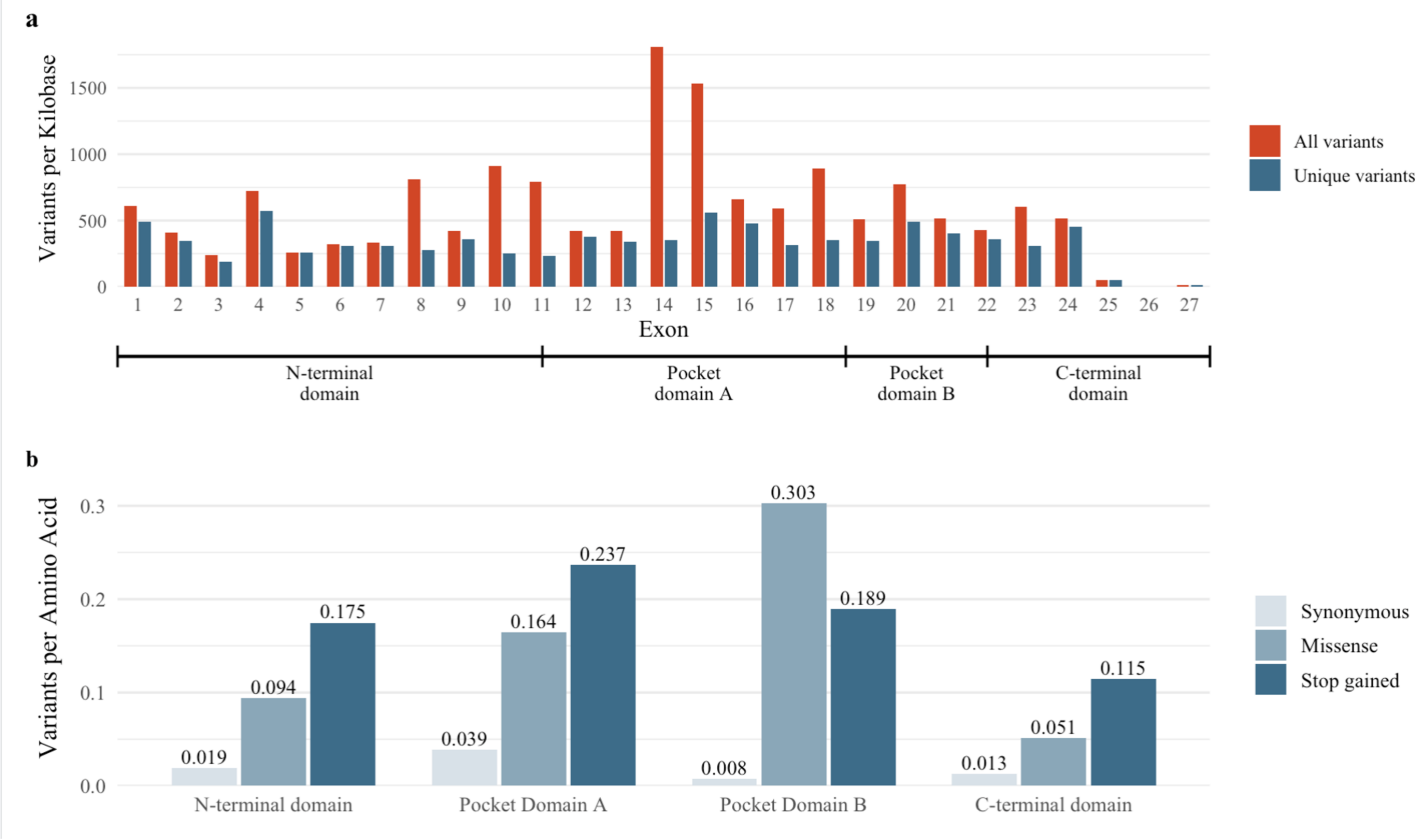
(**a**) Germline RB1 variant density by exon shown as variants per kilobase. *Red bars* display all variants, and *blue bars* indicate unique variants. (**b**) Density of unique germline variants by each single nucleotide variant (SNV) consequence (synonymous, missense, stop gained) in each pRB domain, normalized by protein domain length and by mutational probability of each consequence within each domain.

The distribution of variants in *RB1* was non-uniform across the gene, which can be difficult to interrogate in tabular form. To address this, we developed PANORAMA, a visualization tool for exploring variant data in a genomic and proteomic context that makes *RB1* variant architecture more readily appreciable (Fig. 2). After normalizing for exon length, germline variant density was highest in exons 14 and 15 (>1500 variants/kb) and lowest in exons 25 to 27, including no variants in exon 26 (Fig. 3a). This enrichment was driven by recurrent variants, such as c.1333C>T (p.Arg445*) and c.1363C>T (p.Arg455*) in exon 14 and c.1399C>T in exon 15. When analysis was restricted to unique variants, variant density remained elevated in only exons 4 and 15 (>500 variants/kb).

To understand the functional impact of the identified variants, we next assessed the functional impact on pRB. PANORAMA encodes the molecular consequence of each annotated variant, enabling interrogation of predicted variant effects and their protein consequences. Nonsense (38.6%), frameshift (28.7%), and splicing (16.0%) variants accounted for more than 80% of all germline variants, whereas missense variants comprised 9.2%; non-coding, synonymous, and other less common variant categories together accounted for the remaining 7.5% (Table 2). Simulation of all possible single-nucleotide substitutions across the canonical *RB1* coding sequence revealed that there are more opportunities to create missense (6119) or synonymous (1813) substitutions than to generate nonsense variants (420), yet protein-truncating variants were disproportionately causative among RB cases. Mapping genomic variants to their UniProt^46^ protein domain context demonstrated that per protein variant density for nonsense variants was greatest in the N-terminal, RB_A, and C-terminal regions, whereas missense variants were markedly enriched within the RB_B domain (Fig. 3b). This observation prompted inspection of the x-ray crystallography structure of pRB bound to E2F. A germline variant in RB_B, p.Lys652Asn (listed as pathogenic in LOVD without any supporting functional evidence or ClinVar entry), is located at a residue that forms a hydrogen bond and has a hydrophobic interaction with E2F; whereas, one germline variant in RB_A, p.Lys530Arg (pathogenic in ClinVar and not classified in LOVD), affects a hydrogen bond with E2F. Furthermore, the mean AlphaMissense pathogenicity scores were greatest in RB_B (mean = 0.832), followed by RB_A (mean = 0.639), the C-terminal domain (mean = 0.402), and the N-terminal domain (mean = 0.332).

Next, to assess how clinical designations correlate with computational predictors of pathogenicity, we evaluated germline variants using ClinVar and newer in silico prediction parameters. Of the 1128 unique *RB1* germline variants, only 444 (39.4%) had been previously submitted to ClinVar. Of these, 355 (80.0%) were classified as pathogenic or likely pathogenic, 59 (13.3%) as variants of uncertain significance (VUSs) or conflicting interpretations, and 30 (6.8%) as likely benign or benign, which predominantly were noncoding or synonymous variants in pRB. Only 398 variants had pathogenicity designations by both ClinVar and LOVD, of which there was agreement in 173 (43.5%). The vast majority of non-concordant variants were classified as pathogenic in ClinVar but labeled as a VUS in LOVD (Table 3; for a breakdown of non-concordant ones by variant type, see Supplementary Table S1). Of the 439 variants classified by ClinVar that had CADD scores, those categorized as pathogenic demonstrated higher mean scores (33.3; 95% confidence interval [CI], 32.7–34.0) than VUS (24.0; 95% CI, 22.1–25.8) or benign variants (10.5; 95% CI, 7.2–13.8) (Table 4^5^). When evaluating missense-specific predictions with alpha missense, the majority of the 121 germline missense variants were classified as likely pathogenic (*n* = 65, 53.2%), with the remainder designated as likely benign (*n* = 49, 40.2%) or ambiguous (*n* = 8, 6.6%). When we evaluated both CADD scores and AlphaMissense predictions, we found that together they may have some further discriminatory elements, as none of the likely pathogenic variants had a CADD score below 20. Nine variants classified as likely benign by AlphaMissense had CADD scores < 20, and none was pathogenic in ClinVar. Forty likely benign variants, however, had CADD scores > 20 (Table 5). Finally, we examined the 19 germline synonymous *RB1* variants with synVep to assess predicted effect on splicing, mRNA stability, or translation efficiency. Four of the 19 synonymous variants were classified as pathogenic or likely pathogenic in ClinVar and showed elevated CADD (21.3–26.0) and synVep (0.68–0.95) scores, consistent with their proximity to splice sites. Among the remaining synonymous variants, seven had synVep scores > 0.5, suggesting a possible functional impact (Table 6).

**Table 3.** Comparison of LOVD and ClinVar Variant Classifications

	ClinVar Classifications, *n* (%)					
LOVD Classifications, *n* (%)	Benign	Likely Benign	Uncertain Significance	Likely Pathogenic	Pathogenic	Total
Benign	2 (0.5)	0	0	0	0	2 (0.5)
Likely benign	4 (1)	0	3 (0.8)	0	0	7 (1.8)
Uncertain significance	14 (3.5)	8 (2)	41 (10.3)	31 (7.8)	152 (38.2)	246 (61.8)
Likely pathogenic	0	0	2 (0.5)	1 (0.3)	8 (2)	11 (2.8)
Pathogenic	0	1 (0.3)	1 (0.3)	1 (0.3)	129 (32.4)	132 (33.2)
Total	20 (5)	9 (2.3)	47 (11.8)	33 (8.3)	289 (72.6)	398 (100)

ClinVar classifications of pathogenic/likely pathogenic were grouped into pathogenic, and benign/likely benign were grouped with benign. Variants with conflicting classifications were grouped with variants of uncertain significance.

**Table 4.** ClinVar Classification and CADD Scores

ClinVar Classification	Count, *n* (%)	Mean CADD Score	SD	95% CI
Benign	20 (4.6)	10.51	7.49	7.23–13.79
Likely benign	10 (2.3)	9.34	5.38	6.01–12.67
Uncertain significance	59 (13.4)	24.09	7.25	22.24–25.94
Likely pathogenic	35 (8)	23.91	7.71	21.36–26.46
Pathogenic	315 (71.8)	33.36	5.98	32.7–34.02
Total	439 (100)	29.77	9.11	28.92–30.62

Pathogenic/likely pathogenic classifications were grouped into pathogenic, and benign/likely benign were grouped with benign. Variants with conflicting classifications were grouped with variants of uncertain significance.

**Table 5.** Missense Variants by AlphaMissense Classification and CADD Score

AlphaMissense Classification	CADD > 20, *n*	CADD ≤ 20, *n*	Total, *n*
Likely pathogenic	65	0	65
Ambiguous	8	0	8
Likely benign	40	9	49

Distribution of unique germline missense variants according to AlphaMissense classification and CADD score. Variants were grouped by AlphaMissense predicted pathogenicity class and stratified by CADD score thresholds (>20 vs. ≤20).

**Table 6.** Summary of Germline Synonymous Variants

cDNA	Protein	ClinVar	CADD Score	synVep Score	gnomAD Frequency	SpliceAI Max	Distance to Splice Site
c.861G>A	p.Glu287Glu	Pathogenic	26	0.68	—	0.74	1
c.2211G>A	p.Glu737Glu	Pathogenic	24.4	0.89	—	0.62	1
c.939G>A	p.Glu313Glu	Pathogenic/likely pathogenic	22.4	0.74	—	0.98	1
c.1332G>A	p.Gln444Gln	Pathogenic	21.3	0.95	—	0.89	1
c.1498A>C	p.Arg500Arg	Uncertain significance	18.01	1	0.00000	0	1
c.1335A>C	p.Arg445Arg	—	12.52	0.99	—	0	3
c.1215C>T	p.Asn405Asn	Likely benign	12.39	0.47	0.00002	0.01	1
c.571C>T	p.Leu191Leu	Benign/likely benign	11.17	0.65	0.00016	0	—
c.1596C>T	p.Ile532Ile	Benign/likely benign	11.04	0.1	0.00002	0.01	—
c.1206C>T	p.Ser402Ser	Conflicting classifications	10.94	0.15	0.00001	0.03	10
c.1770T>C	p.Cys590Cys	Benign/likely benign	10.87	0.94	0.00047	0	—
c.1479T>C	p.Val493Val	—	8.51	0.98	0.00000	0.01	20
c.207T>C	p.His69His	Likely benign	8.49	0.39	0.00000	0	—
c.612A>G	p.Glu204Glu	—	7.79	0.85	—	0.06	5
c.42C>T	p.Ala14Ala	Benign/likely benign	7.58	0.04	0.00302	0	—
c.291A>G	p.Glu97Glu	Likely benign	7.03	0.84	0.00000	0	—
c.2455C>T	p.Leu819Leu	Benign/likely benign	5.04	0.25	0.00009	0.01	—
c.1140C>T	p.Asn380Asn	Benign/likely benign	5	0.15	0.00024	0.02	13
c.2463A>G	p.Thr821Thr	Benign/likely benign	0.83	0.43	0.00007	0	—

Finally, to connect these molecular and computational findings with clinically relevant features of retinoblastoma, we evaluated associations between *RB1* variant characteristics and tumor laterality. Germline origin was strongly associated with bilateral disease (χ^2^ = 958.76, degrees of freedom [*df*] = 1, *P* < 0.001), with 81.2% (95% CI, 79.2–83.1) of germline variants associated with bilateral tumors compared with 10.7% (95% CI, 8.4–13.3) of somatic variants. Given that biallelic germline RB1 loss is embryonically lethal, we assumed a one-to-one correspondence between patients and germline variants for germline-specific analyses and therefore performed these analyses at the variant level. The ability to integrate variant and clinical data in PANORAMA demonstrated that truncating variants, comprised of nonsense and frameshift changes, represented approximately 70% of bilateral cases compared with 40% of unilateral cases. Missense variants were more common in unilateral disease (22.1% vs. 7.9% in bilateral), and noncoding variants showed a similar pattern, with higher representation in unilateral cases (9.0% vs. 2.6% in bilateral) (Table 7). However, because these are univariate associations, the relationship between molecular consequence and laterality may be influenced by correlated factors such as germline versus somatic origin. Variant prediction scores revealed that a higher mean CADD score (33.47; 95% CI, 33.12–33.83) was associated with bilateral disease compared to those in unilateral disease (27.63; 95% CI, 26.45–28.81). No specific recurrent variant was significantly associated with laterality after Bonferroni correction for multiple statistical tests (*P* < 4.4 × 10^−5^).

**Table 7.** Variant Consequence Versus Tumor Laterality

Variant Consequence	Unilateral, *n* (%)	Bilateral, *n* (%)	Total, *n*
stop_gained	54 (18.7)	458 (36.6)	512
frameshift	62 (21.5)	413 (33.0)	475
splice_site	48 (16.6)	213 (17.0)	261
missense	64 (22.1)	99 (7.9)	163
intron	26 (9.0)	32 (2.6)	58
inframe_variant	14 (4.8)	16 (1.3)	30
synonymous	11 (3.8)	8 (0.6)	19
5_prime_UTR	8 (2.8)	9 (0.7)	17
large_deletion	1 (0.3)	2 (0.2)	3
start_lost	1 (0.3)	—	1
Total	289 (100.0)	1250 (100.0)	1539

## Discussion

Genomic variants in *RB1* lead to the most common intraocular cancer of childhood, and germline *RB1* variants predispose to extraocular secondary malignancies that carry substantial long-term morbidity and mortality. Although next-generation sequencing and the broader adoption of genetic testing in RB have increased recognition of specific variants that drive disease,^47^ these data remain fragmented across databases and cohort-specific publications. Existing repositories present aggregated variants in tabular or genome-only formats, which restricts visualization of how variants map across the gene and affect the structure and function of pRB. This work addressed these gaps by integrating and standardizing one of the largest curated collections of *RB1* variants in patients with RB on a single reference transcript and genome build to develop PANORAMA, an interactive visualization tool that integrates genomic position, molecular consequence and protein domain impact, and available clinical annotations. More importantly, we expect this tool to transform *RB1* variant curation from static tables into a dynamic evolving repository, as users can both explore the existing landscape and upload additional datasets to expand the variant profile of *RB1* in RB.

Consistent with previous studies,^48^ the aggregated dataset was enriched for germline variants, 72% of which had no familial history of RB, supporting the observation that the majority of germline *RB1* variants arise de novo. Normalization for exon length and restriction to unique variants was able to refine previous indications of hotspot regions^9^^–^^12^ to demonstrate that exons 14 and 15 had a higher variant burden with sparing of exons 25 to 27. The ability to seamlessly view the impact genomic variants have on proteomic architecture with PANORAMA confirmed that variant density is more concentrated in the pocket domain of pRB, consistent with its essential role in tumor suppressor activity.^49^ Examination of the molecular consequences of the curated variants on pRB reinforced that protein-truncating variants are the predominant driver of *RB1*-associated disease. Nonsense, frameshift, and canonical splice-site changes together accounted for more than 80% of germline alterations, despite substantially higher mutational opportunity for missense or synonymous changes. This highlights the strong selective pressure for loss-of-function alleles in RB tumorigenesis and is consistent with prior reports in hereditary RB cohorts where truncating alterations accounted from 57% to 78% of cases.^9^^,^^11^^,^^50^^,^^51^ Our analysis supports this pattern, and molecular consequences can be explored directly in PANORAMA, assisting in revealing structure and function relationships. Mapping variants to pRB domains further clarified domain-specific tolerance to alteration. Although missense variants were less common overall, they were enriched within the RB_B domain, which is a highly conserved subdomain and is necessary and sufficient for the transcriptional repressor activity of pRB.^49^^,^^52^ This finding is supported by evolutionary data^53^ and functional studies^54^ indicating that RB_B is particularly susceptible to pathogenic missense change. A limitation of this aggregated database is alternative *RB1* inactivation mechanisms such as structural variation, copy-number alteration, copy-neutral loss of heterozygosity, and promoter hypermethylation that are biologically important, were only partially captured in the public datasets available for this study, and were absent from the COSMIC retinoblastoma subset.

Variant classification analyses highlighted both the value and the limitations of existing interpretation frameworks. Only a minority of the germline variants in this dataset had corresponding entries in ClinVar, and, among those that did, concordance between ClinVar and LOVD was modest. When examining the discordant entries, we found in cases such as those variants that were noted to be benign in ClinVar and were thought to be causative from case-level data but were primarily due to noncoding or synonymous changes. Such discrepancies likely reflect differences in submission practices, evidence weighting, and update frequency between databases rather than true disagreement on variant effect. The use of variant predictor tools, such as CADD, demonstrated that more severe CADD scores generally aligned with pathogenic ClinVar assignments, whereas benign or likely benign variants generally had lower scores, which is consistent with its observed ability to distinguish their potential role in retinal diseases.^35^ Other tools such as AlphaMissense and synVep had varying levels of concordance with ClinVar, which reflects the need to interpret in silico scores with caution.

Finally, despite limited phenotypic annotation, observed genotype–phenotype relationships were consistent with established clinical patterns. Germline variants were more strongly associated with bilateral disease,^48^ and truncating variants were more frequent in bilateral cases, whereas missense and noncoding variants were relatively enriched in unilateral disease.^11^ This pattern is in line with prior descriptions of low-penetrance *RB1* alleles, in which missense, splice-altering, or promoter variants can produce unilateral tumors or retinomas and show variable expressivity within families.^55^ This is supported by the higher predicted deleteriousness scores of variants seen in bilateral disease cases. Furthermore, the lack of an individual variant driving specific disease suggests that laterality and disease severity are more likely associated with variant class than with specific alleles.

Parent-of-origin effects is an emerging biomarker but its role in RB was limited by sparse annotation of a family history of disease. Differential methylation at the CpG85 region in intron 2 is known to create an imprinting-like effect at *RB1*, and several studies have proposed that parent-of-origin can modify disease severity in specific contexts, with variants that reside on the unmethylated (paternal) allele exhibiting worse disease features compared to those on the methylated (maternal) allele.^13^^,^^14^^,^^56^ To address this gap, PANORAMA incorporates a haplotype-resolved track for phased variants and allele-specific methylation, providing a framework for future analyses as long-read sequencing becomes more widely applied in RB cohorts.

Several strengths of this work warrant emphasis. First, the dataset harmonizes variants across LOVD2, LOVD3, COSMIC, and cohort studies on a single reference transcript (NM_000321.3) and genome build (GRCh38), enabling direct comparisons that were previously difficult because of inconsistent nomenclature and coordinate systems. Second, the resource is explicitly designed to be interactive. Users can upload their own variant lists, align them to the aggregated *RB1* landscape, and immediately assess how their cohort compares. This functionality shifts *RB1* variant curation from a static, table-based format to a visual, gene-centric resource, which is particularly valuable in a gene where biologically important variants span coding exons, splice junctions, and noncoding regions.

This dataset is inherently reliant on the accuracy and completeness of submitter annotations. Phenotypic annotations such as age at diagnosis, tumor burden, treatment, and second malignancies are provided for only a small number of entries, constraining a more granular genotype–phenotype analysis. The data are also likely biased toward individuals and families who underwent clinical genetic testing, were enrolled in research cohorts, or had their data deposited in public databases, likely resulting in overrepresentation of large pedigrees, bilateral cases, and certain geographic or ethnic groups. Large structural variants and copy-number changes are only partially captured. Finally, although we attempted to reconcile and standardize fields across LOVD versions and publications, residual inconsistencies in how submitters recorded variant origin, family history, or mosaic status likely remain.

Expansion of the dataset to include additional cohorts, especially from underrepresented populations and from large sequencing efforts, would improve the generalizability of observed patterns and allow for population-specific analyses. Integration of long-read phasing and allele-specific methylation would allow for analysis of the *RB1* spectrum of variation on a haplotype basis, which may reveal differences in variant haplotype distribution and support mechanisms for the established parent-of-origin effect. Planned tool improvements include allowing for multiple equivalent annotations of coding or protein changes that correspond to a variant to improve searchability of the dataset. Embedding such capabilities into PANORAMA would move it closer to a practical clinical support tool for variant interpretation while the underlying dataset would remain a foundation for investigation of mechanisms of *RB1* disruption driving RB.

## Supplementary Material

Supplement 1
