# Supplementary material for: Integrative Genomic Mapping and Visualization From Curated Public Datasets Reveals Germline RB1 Variant Diversity in Retinoblastoma
Source: Invest Ophthalmol Vis Sci. 2026 Jul 16;67(8):40. doi: 10.1167/iovs.67.8.40 (PMC13387271; doi:10.1167/iovs.67.8.40)
Supplement: Supplement 1 [file iovs-67-8-40_s001.docx]

**Supplemental Table 1. ClinVar and LOVD Classification by Variant Consequence**

|  | **Variant Consequence** | | | | | | | | | |
| --- | --- | --- | --- | --- | --- | --- | --- | --- | --- | --- |
| **Classification** | **Stop gained** | **Frameshift** | **Splice site** | **Missense** | **Intron** | **Inframe variant** | **Synonymous** | **5’ UTR** | **Start lost** | **Total** |
| **ClinVar Classification** |  |  |  |  |  |  |  |  |  |  |
| Benign | 0 | 0 | 0 | 5 | 7 | 1 | 7 | 0 | 0 | 20 |
| Likely benign | 0 | 0 | 1 | 0 | 6 | 0 | 3 | 0 | 0 | 10 |
| Uncertain significance | 0 | 1 | 8 | 39 | 5 | 3 | 2 | 1 | 0 | 59 |
| Likely pathogenic | 0 | 2 | 8 | 8 | 11 | 0 | 0 | 5 | 1 | 35 |
| Pathogenic | 95 | 110 | 85 | 17 | 5 | 1 | 4 | 2 | 0 | 319 |
| **Total** | **95** | **113** | **102** | **69** | **34** | **5** | **16** | **8** | **1** | **443** |
| **LOVD Classification** |  |  |  |  |  |  |  |  |  |  |
| Benign | 0 | 0 | 0 | 0 | 1 | 0 | 1 | 0 | 0 | 2 |
| Likely benign | 0 | 0 | 0 | 4 | 1 | 3 | 1 | 0 | 0 | 9 |
| Uncertain significance | 43 | 183 | 150 | 92 | 49 | 18 | 16 | 13 | 2 | 566 |
| Likely pathogenic | 2 | 0 | 2 | 6 | 1 | 0 | 0 | 0 | 0 | 11 |
| Pathogenic | 96 | 283 | 14 | 5 | 2 | 2 | 0 | 0 | 0 | 402 |
| **Total** | **141** | **466** | **166** | **107** | **54** | **23** | **18** | **13** | **2** | **990** |
| **Unclassified** | 11 | 38 | 10 | 7 | 5 | 3 | 0 | 2 | 0 | 76 |
